# Trans‐spinal magnetic stimulation induces co‐activation of the diaphragm and biceps in healthy subjects

**DOI:** 10.14814/phy2.15941

**Published:** 2024-02-07

**Authors:** Ming‐Yue Ren, Li‐Min Liou, Stéphane Vinit, Kun‐Ze Lee

**Affiliations:** ^1^ Department of Biological Sciences National Sun Yat‐sen University Kaohsiung Taiwan; ^2^ Department of Neurology Kaohsiung Medical University Hospital, Kaohsiung Medical University Kaohsiung Taiwan; ^3^ Department of Neurology, School of Medicine, College of Medicine Kaohsiung Medical University Kaohsiung Taiwan; ^4^ Graduate Institute of Clinical Medicine, College of Medicine Kaohsiung Medical University Kaohsiung Taiwan; ^5^ Université Paris‐Saclay, UVSQ, Inserm, END‐ICAP Versailles France; ^6^ Department of Biomedical Science and Environmental Biology Kaohsiung Medical University Kaohsiung Taiwan

**Keywords:** biceps, diaphragm, figure‐of‐eight coil, trans‐spinal magnetic stimulation

## Abstract

The present study was designed to examine the effect of trans‐spinal magnetic stimulation on bilateral respiratory and forelimb muscles in healthy subjects. Two wings of a figure‐of‐eight magnetic coil were placed on the dorsal vertebrae, from the fifth cervical to the second thoracic dorsal vertebra with a center at the seventh cervical vertebra. The surface electromyograms of bilateral diaphragm and biceps were recorded in response to trans‐spinal magnetic stimulation with 20%–100% maximum output of the stimulatory device in male (*n* = 12) and female participants (*n* = 8). Trans‐spinal magnetic stimulation can induce a co‐activation of bilateral diaphragm and biceps when the stimulation intensity is above 60%. The onset latency was comparable between the left and right sides of the muscles, suggesting bilateral muscles could be simultaneously activated by trans‐spinal magnetic stimulation. In addition, the intensity–response curve of the biceps was shifted upward compared with that of the diaphragm in males, indicating that the responsiveness of the biceps was greater than that of the diaphragm. This study demonstrated the feasibility of utilizing trans‐spinal magnetic stimulation to co‐activate the bilateral diaphragm and biceps. We proposed that this stimulatory configuration can be an efficient approach to activate both respiratory and forelimb muscles.

## Graphical abstract

Trans‐spinal magnetic stimulation is a feasible method to activate the diaphragm and biceps and produce the motor evoked potential recorded by surface electromyogram. Coactivation of bilateral diaphragm and biceps could be induced by trans‐spinal magnetic stimulation simultaneously when the intensity is over 60% maximal stimulus output.

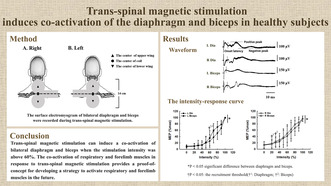

## 1 INTRODUCTION

Magnetic stimulation is a noninvasive method to activate neural tissues through induction of eddy currents using a time‐varying magnetic field. The magnetic coil can be placed on the cortex, spinal cord, and peripheral nerves to induce motor‐evoked potentials of respiratory or forelimb muscles (Bawa et al., 2004; Corfield et al., 1998; Demoule et al., 2003; Hamnegård et al., 1996; Locher et al., 2006; Mills et al., 1995; Ross et al., 2007; Similowski et al., 1996). This approach can be used to examine the excitability of the respiratory and forelimb motor systems under physiological and pathological conditions (Demoule et al., 2003; Hughes et al., 1999; Laviolette et al., 2013; Straus et al., 2004). Moreover, magnetic stimulation with repetitive stimulatory pattern can induce neuroplasticity and modulate motoneuronal excitability (Lefaucheur et al., 2020; Suppa et al., 2022). However, most studies used transcranial magnetic stimulation to investigate one of these motor systems and have not compared the responses of the respiratory and forelimb motor systems together (Boyle et al., 2020; Guenette et al., 2010; Maskill et al., 1991). Several studies have demonstrated that there is a significant interaction between the respiratory and forelimb motor systems. For example, nonrespiratory maneuvers (e.g., biceps curls) can strengthen the inspiratory muscles in healthy subjects by increasing the diaphragm thickness and transdiaphragmatic pressure (DePalo et al., 2004). Similarly, respiratory muscle training can also enhance both respiratory and nonrespiratory motor function (Markov et al., 2001; Randelman et al., 2021). These results suggest that coordination and interaction between respiratory and forelimb motor systems serve an important physiological purpose, thus raising the possibility and significance of activating both motor systems with magnetic stimulation.

Several magnetic stimulatory methods have been used to activate bilateral respiratory and/or limb muscles; however, there are still some concerns of using these approaches. For example, transcranial magnetic stimulation typically actives contralateral muscles with a weaker excitation of ipsilateral muscles (Eldaief et al., 2013). Anterior presternal magnetic stimulation can activate bilateral diaphragm, but the magnetic coil has to be placed on the chest, which may recruit thoracic muscles and influence the heart conducting system (Man et al., 2004). Bilateral anterolateral magnetic phrenic nerve stimulation can specifically and simultaneously activate bilateral diaphragm, but this approach requires two stimulators and two coils (Mador et al., 2002; Man et al., 2004). Accordingly, it is important to establish an efficient and convenient approach to induce co‐activation of bilateral respiratory and forelimb muscles to comprehensively and simultaneously examine the excitability of these two muscles during trans‐spinal magnetic stimulation.

The diaphragm is innervated by phrenic motoneurons, which are located within the third to fifth cervical spinal cord (Keswani & Hollinshead, 1955), and motoneurons within these cervical segments also innervate the biceps (Waxenbaum et al., 2022). It seems reasonable that magnetic stimulation at the cervical spinal cord should be able to activate both diaphragm and biceps. However, there are several different neuroanatomical and neurophysiological properties of these two motor systems. For example, the diaphragm is mainly controlled by the bulbospinal tracts and phrenic nerves, while the biceps is primarily controlled by the corticospinal pathways and musculocutaneous nerves. In addition, activation of the diaphragm is usually automatic while contraction of biceps is generally voluntary. Thus, it is still unclear whether the diaphragm and biceps could be co‐activated when the magnetic field covered the cervical spinal cord. Accordingly, the present study aimed to evaluate whether trans‐spinal magnetic stimulation using a figure‐of‐eight coil covering the dorsal cervical vertebrae can induce co‐activation of bilateral diaphragm and biceps, and compare the response curve of bilateral diaphragm and biceps motor‐evoked potential in healthy subjects. We hypothesized that both diaphragm and biceps could be co‐activated by trans‐spinal magnetic stimulation at high stimulatory intensity, but the differential excitability of these two muscles could be revealed during low to moderate stimulatory intensity.

## 2 METHODS

### 2.1 Participants

All experimental procedures were approved by the Institutional Review Board at Kaohsiung Medical University Hospital (KMUHIRB‐F(I)‐20210039). Twenty healthy adults (male, *n* = 12; female, *n* = 8) free of respiratory and/or neurological disorders were recruited, and all provided written informed consent before participating in the experiment (Table 1). The exclusion criteria included pregnancy, respiratory disorders (e.g., asthma and pneumonia), neurological diseases (e.g., epilepsy, stroke, ankylosing spondylitis, and herniated intervertebral disc), presence of a metal implant, and having undergone spine/cranial surgery (Ren et al., 2022; Rossini et al., 2015).

**TABLE 1:** Anthropometry of participants.

	Males (*n* = 12)	Females (*n* = 8)
Age (years)	21.9 ± 2.3	20.9 ± 1.0
Height (cm)	174.2 ± 6.2	162.3 ± 5.6*
Weight (kg)	66.1 ± 12.8	53.0 ± 6.3*
BMI (kg/m^2^)	21.7 ± 3.6	20.1 ± 1.6

*Note*: Data are mean ± standard deviation.

Abbreviation: BMI: body mass index.

* *p* < 0.05 significantly difference between males and females.

### 2.2 Surface electromyogram recording

Electromyogram (EMG) recordings of bilateral diaphragm and biceps were made using a pair of surface electrodes (HR‐OP37, MKR0716000, Hurev Co., Ltd.) for each muscle. The participant was seated on a chair with their head leaned forward on the headrest. For diaphragm EMGs, the electrodes were placed on the bilateral chest wall between the sixth and eighth intercostal spaces at 2‐cm intervals along the midclavicular line (Verin et al., 2002). Bilateral biceps EMGs were recorded by placing electrodes on the anterior two‐thirds of the arm, between the acromion process and cubital fossa (Singla et al., 2018). A ground surface electrode was placed on the back of the left hand. All electrodes were fixed using tape (#18225, 3 M Nexcare) and connected with a cable (LEADP1026S3, Spes Medica Srl) to a differential A/C amplifier (gain: 1000×, band‐pass filtered: 10–1000 Hz, D360 8‐Channel Patient Amplifier, Digitimer). Participants were asked to take deep inspirations three times after at least 1 min of resting breathing to confirm the inspiratory activity of the diaphragm. Forelimb isometric contractions were performed in all subjects to confirm the activity of the biceps.

### 2.3 Trans‐spinal magnetic stimulation

**FIGURE 1:**
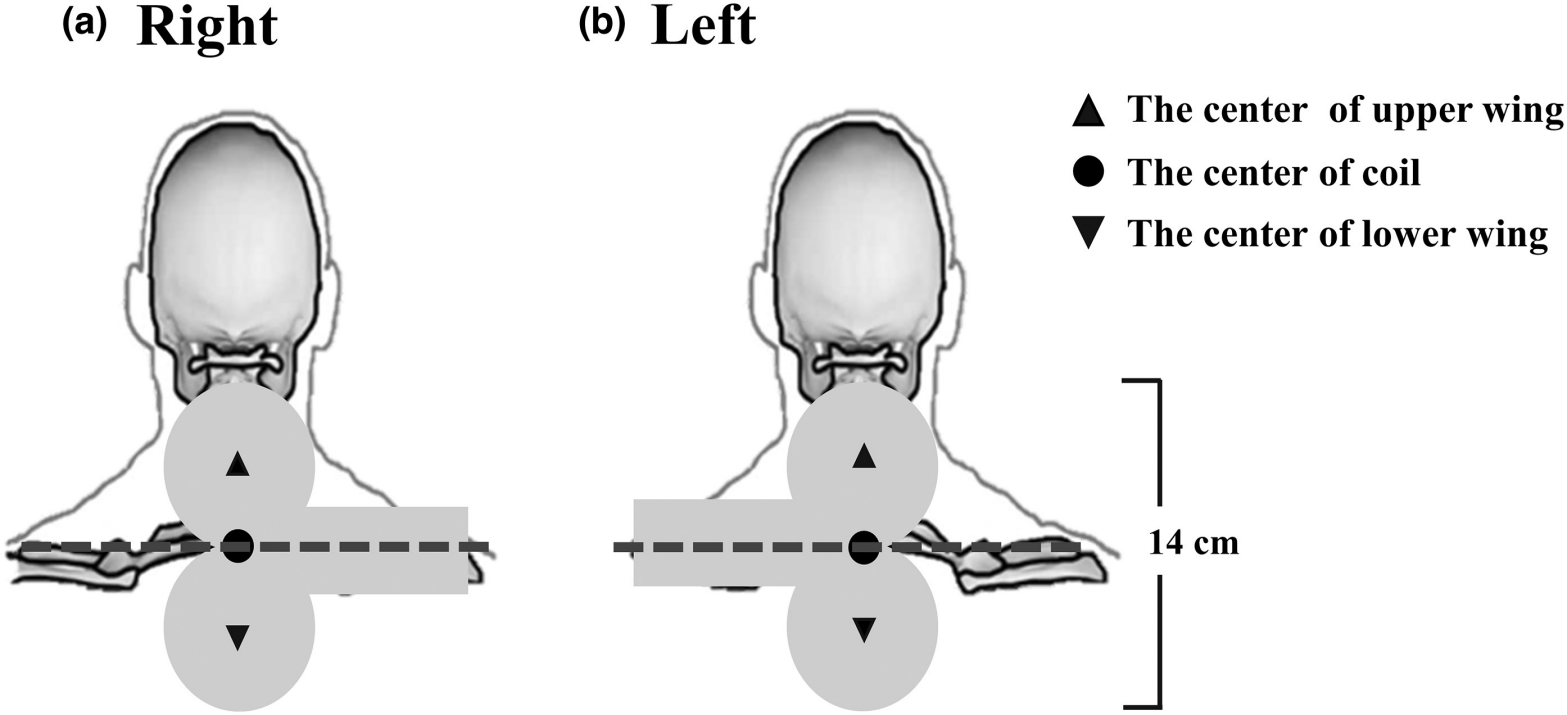
Schematic representation of the spine and coil positioning. The center of the coil was placed on the seventh cervical vertebrae (C7). The two wings of the figure‐of‐eight magnetic coil (MCF‐B65, inner diameter: 35 mm; outer diameter: 75 mm) covered the mid‐cervical to high‐thoracic portion of the spine with its handle toward the right (a) or the left (b) side of the subject. The scale bar is in the right side.

Trans‐spinal magnetic stimulation was conducted using a figure‐of‐eight coil (MCF‐B65, inner diameter: 35 mm; outer diameter: 75 mm; max initial dB/dt: 32 kT/s, peak magnetic flux density: 1.4 T, MagVenture, Inc.) connected to a magnetic stimulator (MagPro R30, MagVenture, Inc.). The coil was placed on the dorsal vertebrae of the participants using a stand (9016B017, MagVenture, Inc.), and the handle of the coil was held to the right (Figure 1a) or left (Figure 1b) at random. The center of the coil was positioned at the seventh cervical vertebral column; this configuration can enable two coil wings to cover the mid‐cervical to the high thoracic portion of the spine. Ten single pulses of magnetic stimulation from 20% to 100% (10% increments with 2‐min intervals) of the maximum stimulator output were applied during end‐expiration, as determined through visual observation by the operator.

### 2.4 Data analysis and statistics

EMG signals from the diaphragm and biceps were digitized using a CED Power 1401 (Cambridge Electronic Design Ltd.) at a sampling rate of 100 kHz (for motor‐evoked potential) using the Spike 2 and Signal software (Cambridge Electronic Design Ltd). The raw EMG signals of all recorded muscles were rectified and smoothed (time constant: 25 ms). The activity of each muscle was defined as the difference between the maximum value measured during muscle contraction (i.e., deep inspiration or forelimb contraction) and the minimum value measured during muscle relaxation (i.e., expiration and forelimb relaxation). The amplitude of the motor‐evoked potential induced by trans‐spinal magnetic stimulation was calculated for all muscles as the peak‐to‐peak value of raw EMG signals and was averaged for each stimulation intensity. Peak‐to‐peak amplitude is defined as the difference between the maximal (i.e., positive peak) and minimal (i.e., negative peak) value of the waveform. These data were presented in terms of absolute voltage and were normalized by a percentage of the maximum response. The onset latency is calculated as significant fluctuation of the waveform compared with the baseline (i.e., before stimulation) during 100% intensity. To prevent interference of the stimulus artifact, the onset latency was analyzed after 2 ms of stimulation. The recruitment threshold means that the motor‐evoked potential is significantly greater than the background signal values at the lowest stimulatory intensity.

The preliminary analysis demonstrated that the placement of the coil handle (i.e., to the right [Figure 1a] or left [Figure 1b]) had no significant effect on the amplitude of normalized motor‐evoked potential in the diaphragm and biceps. We therefore combined the data from both sides to evaluate the effect of trans‐spinal magnetic stimulation.

A paired *t*‐test was used to evaluate the difference in amplitude and latency between the EMG signals from the left to right muscles during either deep inspiration, forelimb contraction, or response to trans‐magnetic stimulation. One‐way repeated measures analysis of variance was used to assess the motor‐evoked potential amplitude among the diaphragm and biceps during different stimulatory intensity of trans‐spinal magnetic stimulation. A *t*‐test was used to compare the onset latency of motor‐evoked potential between male and female subjects. If the data did not pass the normality and equal variance tests, appropriate nonparametric statistical tests such as rank‐based analysis of variance or the Mann–Whitney rank sum test were used to evaluate the experimental effects. All data are expressed as mean ± standard deviation. *p* < 0.05 was considered statistically significant.

## 3 RESULTS

### 3.1 Anthropometry of the participants

Anthropometric data for the 20 participants are provided in Table 1. Age and body mass index were similar between the male and female participants, but height and weight were significantly lower in female than in male participants (*p* < 0.05, Table 1).

### 3.2 Electromyograms of the diaphragm and biceps during deep inspiration and forelimb contraction

**FIGURE 2:**
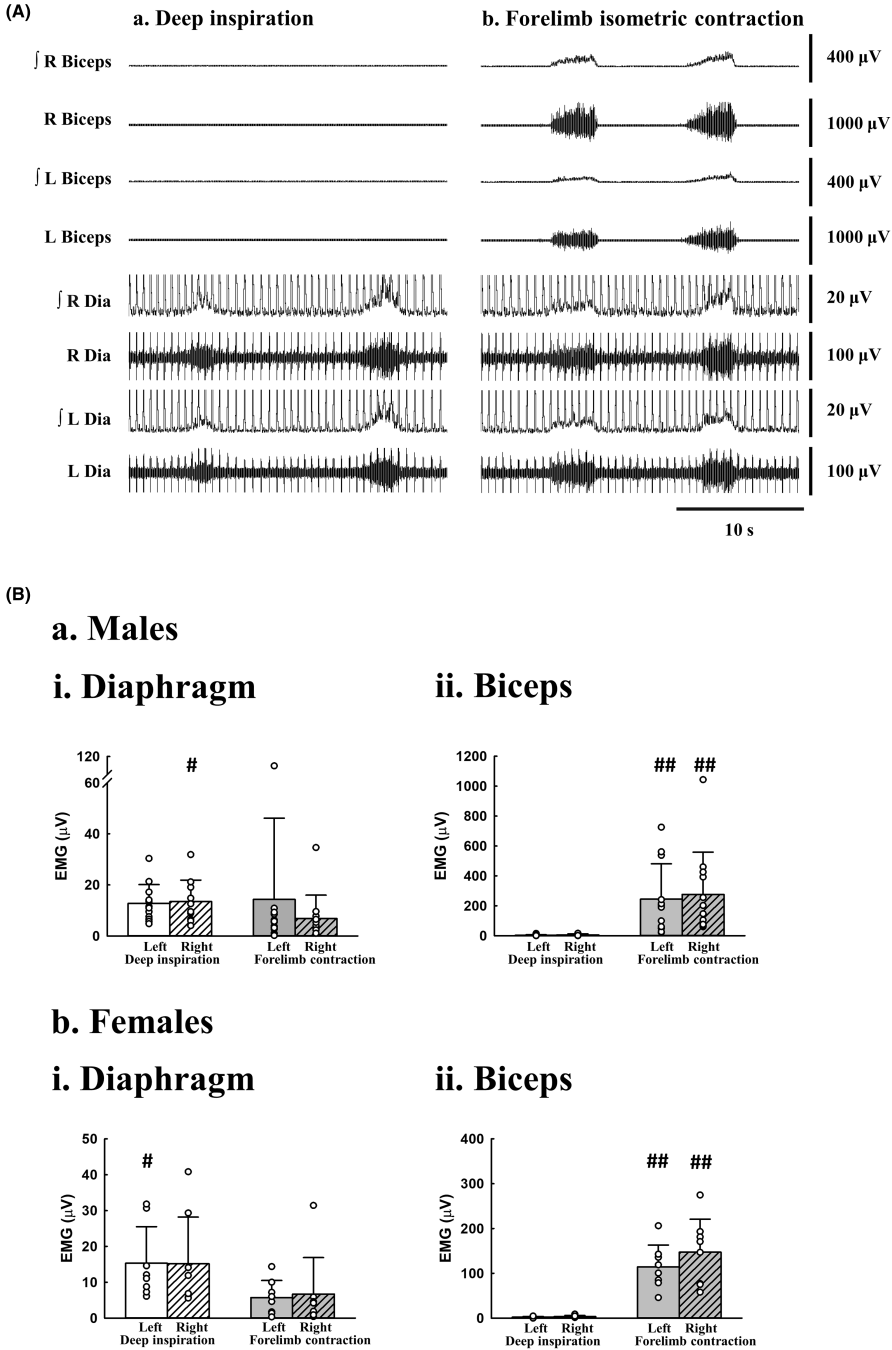
Electromyograms of the diaphragm and biceps. Representative examples of the bilateral diaphragm (Dia) and biceps electromyogram (EMG) during deep inspiration (Aa) and forelimb maximal isometric contraction (Ab) in a subject are shown in panel (A). The data trace is presented as the raw signal and the rectified and smoothed signals (∫) of the right and left muscles. Panel (B) presents the amplitude of the diaphragm (i) and biceps (ii) in both male (a, *n* = 12) and female (b, *n* = 8) subjects. Data are presented as mean + standard deviation (bar chart) and individual data points (white circles). #*p* < 0.05: significant difference between two conditions. L, left muscle; R, right muscle. ^##^ *p* < 0.001: significant difference between two conditions.

Representative examples of the bilateral diaphragm and biceps EMGs obtained during deep inspiration (Figure 2Aa) and forelimb isometric contraction (Figure 2Ab) are shown in Figure 2. Significant inspiratory bursting was observed in the diaphragm, but not the biceps, during deep inspiration (Figure 2Aa). However, a significant increase in burst amplitude was observed in all muscles during forelimb isometric contraction (Figure 2Ab). The amplitude of left and right muscles was comparable during deep inspiration and forelimb contraction in both sexes (Figure 2b). The amplitude of diaphragm EMG was generally greater during deep inspiration than during forelimb contraction in both male (right side, *p* = 0.042, Figure 2Bai) and female (left side, *p* = 0.027, Figure. 2Bbi) subjects. The EMG signal for bilateral biceps showed a greater amplitude during forelimb contraction than during deep inspiration in all subjects (*p* < 0.001, Figure 2Baii,Bbii).

### 3.3 Diaphragmatic motor‐evoked potentials during trans‐spinal magnetic stimulation

**FIGURE 3:**
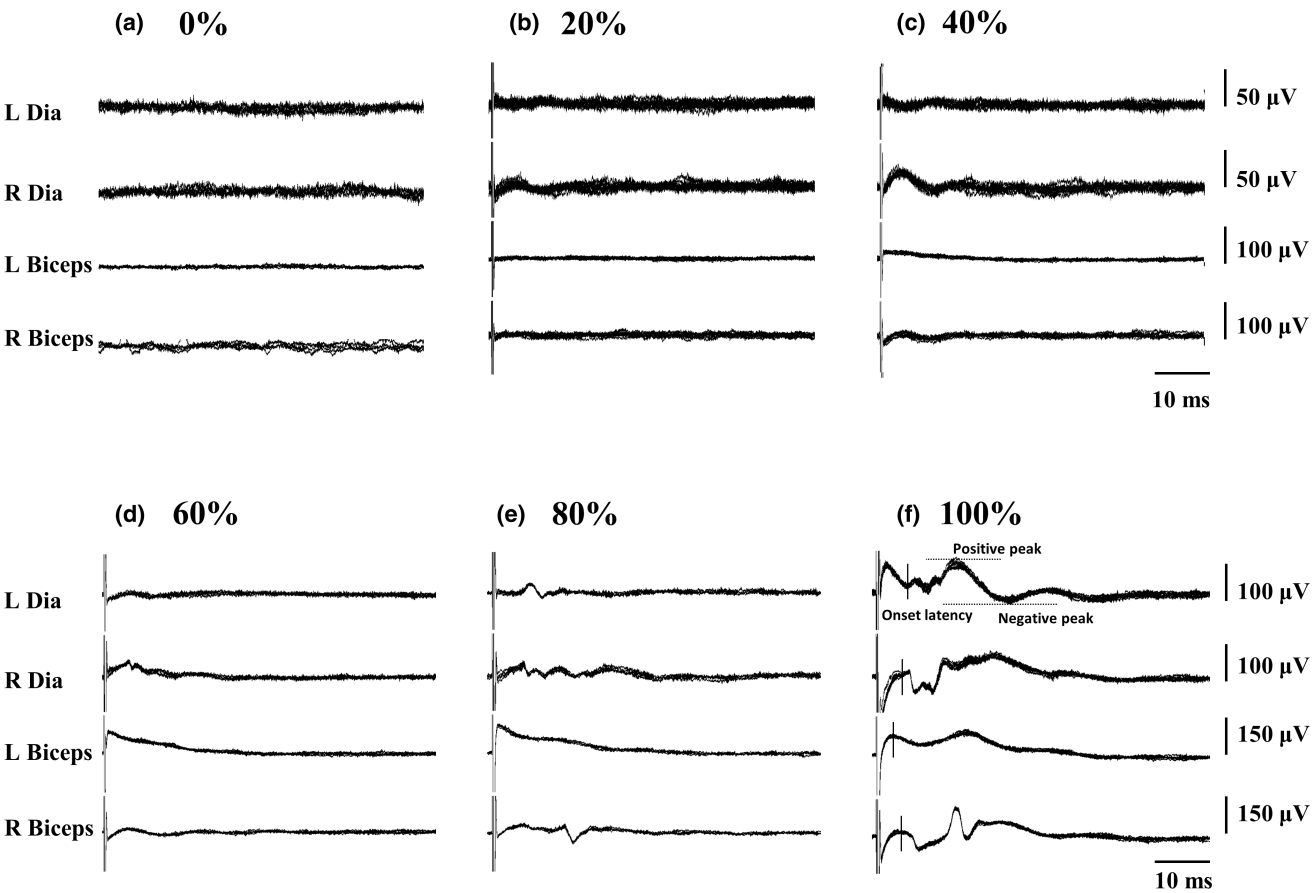
Motor‐evoked potential of the diaphragm and biceps in response to trans‐spinal magnetic stimulation. The figure shows motor‐evoked potential of the bilateral diaphragm (Dia) and biceps in response to 0% (a), 20% (b), 40% (c), 60% (d), 80% (e), and 100% (f) stimulation intensity (% of maximal output of the stimulator). Data traces are superimposed for 10 waveforms in response to each stimulus intensity. The dotted line denotes peak‐to‐peak amplitude, and the black line denotes the onset latency. L, left muscle; R, right muscle.

**FIGURE 4:**
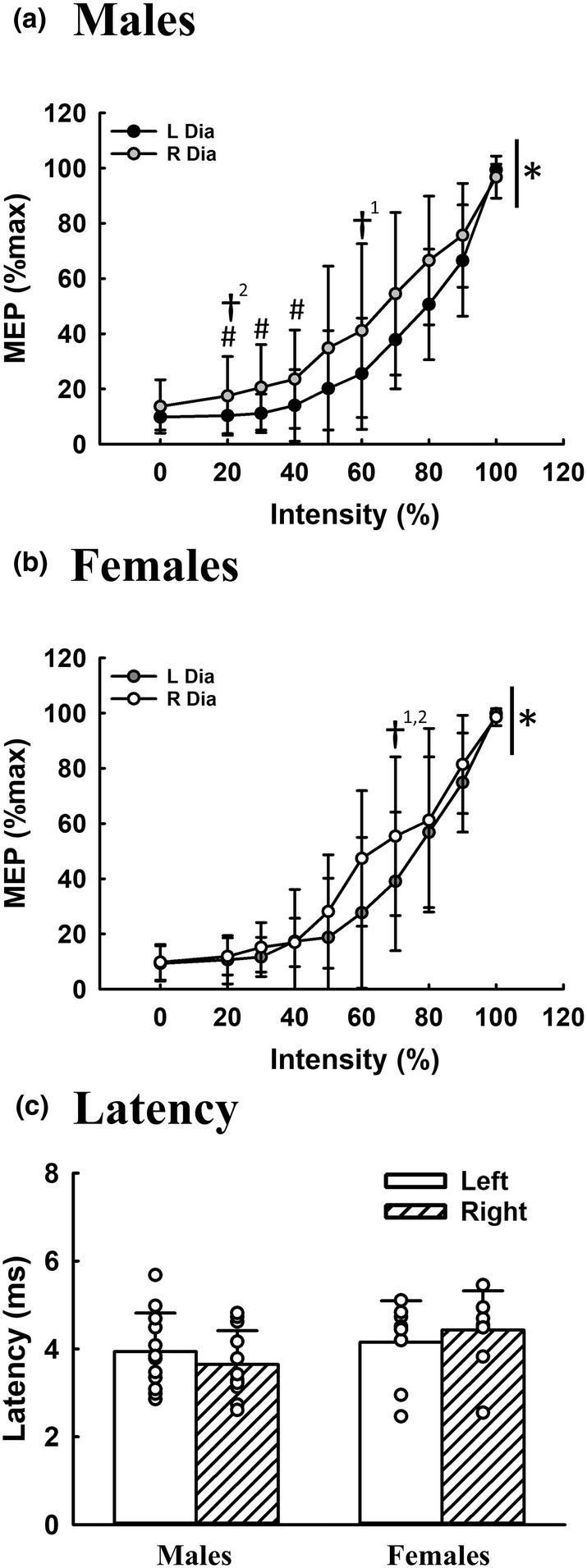
The intensity–response curve and latency of the diaphragmatic motor‐evoked potential in response to trans‐spinal magnetic stimulation. The intensity–response curve of bilateral diaphragmatic motor‐evoked potential in male (a, *n* = 12) and female (b, *n* = 8) subjects are presented as a percentage of the maximal response during trans‐spinal magnetic stimulation. Panel (c) shows the onset latency of diaphragmatic motor‐evoked potential induced by 100% intensity. *p < 0.05: significant difference between the intensity–response curve of the left and right muscles. #*p* < 0.05: significant difference between the response of left and right muscles at a specific intensity. ^†^ *p* < 0.05: the recruitment threshold (†^1^: left side; †^2^: right side) (i.e., the response is significantly greater than the background signal values at the lowest stimulatory intensity).

Representative examples of bilateral diaphragmatic motor‐evoked potential induced by trans‐spinal magnetic stimulation are shown in Figure 3. The peak‐to‐peak amplitudes of motor‐evoked potentials are presented as raw values (μV, Table 2) and as a percentage of the maximum response (%max, Figure 4). The background signal values of the right and left diaphragm during resting state (i.e., 0% stimulation) in male subjects were 9.0 ± 2.6 and 8.2 ± 1.9 μV, respectively. The bilateral diaphragmatic motor‐evoked potential was gradually increased as stimulatory intensity increased. Specifically, the amplitude of the right diaphragmatic motor‐evoked potential (11.7 ± 6.5 μV; 17.5 ± 14.3%max) was significantly higher than the value during the resting state in male subjects when the stimulation intensity increased to 20% (*p* = 0.043, Figure 4a). However, the left diaphragmatic motor‐evoked potential (26.9 ± 35.1 μV; 25.5 ± 20.2%max) in males was significantly induced until the stimulatory intensity was increased to 60% (*p* = 0.024, Figure 4a). The background signal values of the right and left diaphragm during resting state in female subjects were 9.4 ± 2.9 μV (9.8 ± 6.5%max) and 9.2 ± 2.4 μV (9.3 ± 6.4%max), respectively. The amplitude of right and left diaphragmatic motor‐evoked potential (right: 44.9 ± 11.7 μV, 47.4 ± 24.5%max; left: 26.4 ± 21.3 μV, 27.7 ± 27.3%max) was significantly higher than the value during the resting state in female subjects when the stimulation intensity increased to 60% (*p* < 0.001, Figure 4b).The amplitude of bilateral diaphragmatic motor‐evoked potential in both sexes could still be augmented when the stimulatory intensity increased from 70% to 100%. The diaphragmatic motor‐evoked potential amplitude at 100% intensity was significantly greater than the response at 90% intensity in bilateral diaphragm of males and females, suggesting that the diaphragmatic motor‐evoked potential may not have reached the plateau response during 100% trans‐spinal magnetic stimulation (Figure 4a,b; Table 2). In addition, the intensity–response curve of the right diaphragm was shifted upward compared with the curve of the left diaphragm in both sexes. These results suggested that excitability and responsiveness of the right diaphragm may be greater than those of the left diaphragm in response to trans‐spinal magnetic stimulation (*p* < 0.001, Figure 4a,b). However, the onset latency of diaphragmatic motor‐evoked potential in response to 100% intensity was similar between the left and right diaphragm, suggesting trans‐spinal magnetic stimulation can simultaneously activate bilateral diaphragm (Figure 4c).

**TABLE 2:** The raw amplitude of motor‐evoked potentials of the diaphragm and biceps in male and female subjects.

Intensity (%)	Diaphragm (μV)						Biceps (μV)					
	Left			Right			Left			Right		
	Mean	SD	Median	Mean	SD	Median	Mean	SD	Median	Mean	SD	Median
Males												
0	8.2	1.9	8.5	9.0	2.6	9.9	8.0	6.4	6.2	8.5	4.5	8.1
20	8.7^#^	2.8	8.4	11.7^†^	6.5	9.9	8.2	3.5	7.8	9.7†	3.9	8.6
30	9.5^#^	4.6	10.0	15.5	9.5	13.6	8.9^#^	5.0	7.6	14.6^†^	5.1	11.6
40	11.8	9.1	10.1	17.5	9.6	15.6	11.6^#^	6.3	9.2	17.5	4.6	14.8
50	23.2	36.7	10.1	24.7	12.7	23.3	22.8	16.7	21.5	26.3	10.7	25.0
60	26.9^†^	35.1	14.4	30.5	14.8	32.3	32.9^†^	19.0	32.3	62.3	114.7	28.6
70	44.0	43.5	27.1	42.4	13.4	39.6	60.9	28.9	54.1	104.2	235.3	36.9
80	58.0	55.2	36.8	66.1	47.2	49.7	104.3	138.1	66.9	131.8	276.1	52.6
90	69.6	49.8	50.0	83.9	75.7	55.9	115.7	161.9	71.5	137.6	266.0	56.5
100	107.9	67.1	88.4	121.0	109.9	88.9	147.2	192.0	83.6	170.8	315.5	56.9
Females												
0	9.2	2.4	9.2	9.4	2.9	9.0	9.6	4.0	9.6	9.1	2.7	9.3
20	9.6	1.4	9.9	12.4^†^	5.6	10.7	8.8	2.5	8.7	10.1	4.9	9.2
30	11.7	2.9	12.6	15.0	5.3	15.7	20.3^†^	19.0	13.2	17.1^†^	10.4	15.5
40	16.9	15.3	11.7	17.4	7.1	17.4	24.3	20.1	17.3	26.1	14.8	23.4
50	19.7	18.6	15.3	27.6	13.9	26.4	29.5	23.3	26.2	27.8	15.4	23.2
60	26.4^†#^	21.3	19.4	44.9	11.7	48.1	41.2	19.4	33.9	32.0	16.7	28.7
70	39.8	18.0	36.7	52.9	12.4	58.7	53.8	39.5	38.7	39.7	20.3	30.5
80	61.4	23.4	71.5	57.5	15.1	62.2	64.8	58.8	43.5	46.8	28.1	34.4
90	96.6	53.6	97.8	99.1	53.5	85.7	115.8	105.1	63.8	79.8	59.1	69.6
100	130.6	71.1	116.3	130.4	84.9	105.1	158.6	145.0	102.5	125.5	86.3	138.0

Abbreviation: SD: standard deviation.

^†^ *p* < 0.05, MEP induced by this intensity is greater than that induced by 0% intensity.

^*#*^ *p* < 0.05, left muscle versus right muscle.

### 3.4 Biceps motor‐evoked potentials during trans‐spinal magnetic stimulation

**FIGURE 5:**
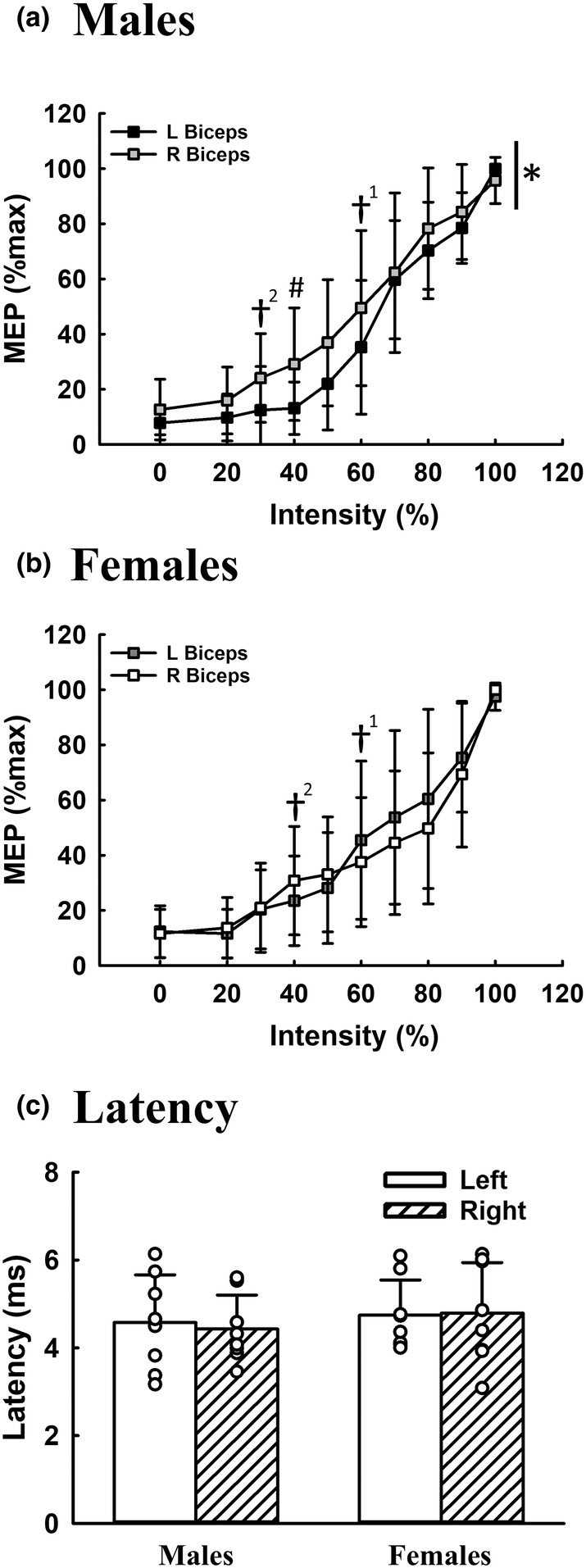
The intensity–response curve and latency of the biceps motor‐evoked potential in response to trans‐spinal magnetic stimulation. The intensity–response curve of bilateral biceps motor‐evoked potential in male (a, *n* = 12) and female (b, *n* = 8) subjects are presented as a percentage of the maximal response during trans‐spinal magnetic stimulation. Panel (c) shows the onset latency of biceps motor‐evoked potential induced by 100% intensity. *p < 0.05: significant difference between the intensity–response curve of the left and right muscles. ^#^ *p* < 0.05: significant difference between the response of left and right muscles at a specific intensity. ^†^ *p* < 0.05: the recruitment threshold (†^1^: left side; †^2^: right side) (i.e., the response is significantly greater than the background signal values at the lowest stimulatory intensity).

Representative examples of bilateral biceps motor‐evoked potential induced by trans‐spinal magnetic stimulation are shown in Figure 3. Intensity‐dependent response of biceps motor‐evoked potential was observed in both male and female subjects. Specifically, the amplitude of the motor‐evoked potential in the right biceps at 30% intensity reached 14.6 ± 5.1 μV (24.1 ± 16.1%max), which was significantly higher than the background value (8.5 ± 4.5 μV; 12.7 ± 11.0%max) in male subjects (*p* < 0.001, Figure 5a). Notably, a significant left biceps motor‐evoked potential could only be observed until the stimulatory intensity reached 60% (32.9 ± 19.0 μV; 35.2 ± 24.3%max) in males (*p* = 0.001, Figure 5a). Regarding female subjects, the right biceps motor‐evoked potential was significantly greater during 40% stimulatory intensity (26.1 ± 14.8 μV, 30.8 ± 19.7%max, *p* = 0.015) than during the resting state (9.1 ± 2.7 μV, 11.6 ± 8.8%max) (Figure 5b), while the left biceps motor‐evoked potential was significantly greater during 60% stimulatory intensity (41.2 ± 19.4 μV, 45.5 ± 28.7%max, *p* = 0.001) than during the resting state (9.6 ± 4.0 μV, 12.3 ± 9.4%max) in females (Figure 5b). Hence, the recruitment thresholds of right and left biceps in females were 40% and 60%, respectively. The biceps motor‐evoked potential gradually augmented when the stimulatory intensity increased from 60% to 100%, and the amplitude usually reached the maximal value at 100% intensity in both side muscles (Figure 5a,b). The onset latency of bilateral biceps was comparable in both male and female subjects, indicating trans‐spinal magnetic stimulation can induce co‐activation of bilateral biceps (Figure 5c). Moreover, the intensity–response curve of the right biceps was shifted upward compared with the curve of the left biceps in males (*p* < 0.001, Figure 5a), indicating that excitability and responsiveness are higher in the right biceps than the left in response to trans‐spinal magnetic stimulation.

### 3.5 Co‐activation of diaphragm and biceps motor‐evoked potential during trans‐spinal magnetic stimulation

**FIGURE 6:**
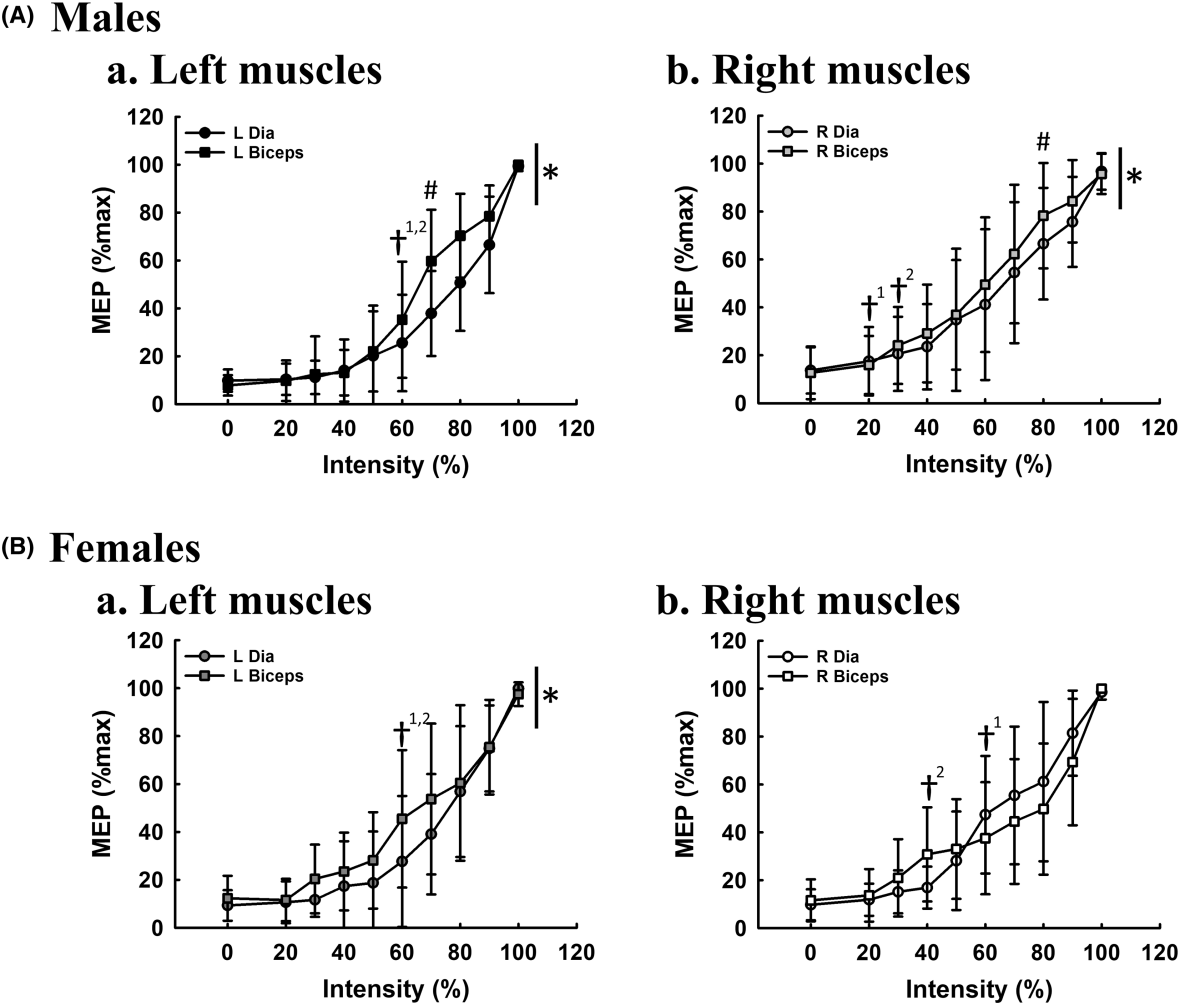
Comparison of the intensity–response curve between diaphragm and biceps. The intensity–response curve of the left (a) and right (b) diaphragm (Dia) and biceps, was compared in male (A, *n* = 12) and female (B, *n* = 8) subjects. *p < 0.05: significant difference between the intensity–response curve of diaphragm and biceps. ^#^ *p* < 0.05: significant difference between the response of diaphragm and biceps at specific intensity. ^†^ *p* < 0.05: the recruitment threshold (†^1^: diaphragm; †^2^: biceps) (i.e., the response is significantly greater than the background signal values at the lowest stimulatory intensity).

The intensity–response curve between the diaphragm and biceps motor‐evoked potential is demonstrated in Figure 6. Trans‐spinal magnetic stimulation can induce an intensity‐dependent response of both diaphragm and biceps motor‐evoked potential from 20% to 100% intensity. The recruitment threshold is similar between the diaphragm and biceps in left muscles (Figures 6Aa,Ba). However, we noticed that the intensity–response curve of biceps was shifted upward relative to that of the diaphragm on both sides in male participants (left, *p* = 0.002; right, *p* = 0.009; Figure 6A), indicating that excitability and responsiveness are higher in the biceps than in the diaphragm in response to trans‐spinal magnetic stimulation. This differential diaphragm versus biceps response was also observed in the left side of females (*p* = 0.001, Figure 6Ba). In general, when the intensity is over 60%, the bilateral diaphragm and biceps would be co‐activated in both males and females.

## 4 DISCUSSION

The present study demonstrates that trans‐spinal magnetic stimulation using a figure‐of‐eight coil with two wings covering the mid‐cervical to the upper thoracic portion of the spinal cord can induce motor‐evoked potentials in bilateral diaphragm and biceps in subjects of both sexes. Moreover, during trans‐spinal magnetic stimulation, the intensity–response curve for the right diaphragm was shifted upward compared with that of the left diaphragm in both male and female subjects, indicating that the responsiveness of the diaphragm is distinct between the right and left sides. Furthermore, the responsiveness during trans‐spinal magnetic stimulation in male subjects is usually higher in the biceps than in the diaphragm. These results suggest that the use of the current trans‐spinal magnetic stimulatory configuration to co‐activate the bilateral diaphragm and biceps is feasible. We propose that this approach can be used in a clinical context as an efficient and convenient strategy for the activation of both respiratory and forelimb muscles.

### 4.1 Critique of method

Several methodological limitations, including the specificity of surface EMG electrodes, the time point for trans‐spinal magnetic stimulation, and the influence of neck curvature on the contact between the coil and the vertebrae, have been discussed extensively in our recent report (Ren et al., 2022). However, there are additional issues that require further discussion. First, the two wings of the coil covered the vertebrae from the mid‐cervical to high thoracic segments (Figure 1). This stimulatory configuration can extensively activate the spinal cord, spinal roots, and spinal nerves from the cervical to the thoracic level; therefore, the motor‐evoked potentials induced in the present study are a summation response from the stimulation of the mid‐cervical to high thoracic spinal cord. Second, coil orientation has a substantial influence on the effectiveness of transcranial magnetic stimulation in both humans and animals (Janssen et al., 2015; Richter et al., 2013; Vinit et al., 2014). Although this study demonstrated that the amplitude of the normalized motor‐evoked potentials was not affected by whether the coil handle was held to the right or left of the spine, we cannot exclude the possibility that positioning the coil at other angle might produce a differential response between left‐ and right‐side muscles. Future studies are needed to comprehensively examine the effect of coil orientation on trans‐spinal magnetic stimulation‐induced motor‐evoked potentials. Third, the neck length may be different between sexes because males are significantly taller than females. As different neck lengths may influence the number of spinal segments covered by the magnetic field, we proposed that trans‐spinal magnetic stimulation may activate more spinal segments in female subjects and cause the differential response compared with male subjects.

### 4.2 Co‐activation of bilateral muscles during trans‐spinal magnetic stimulation

Transcranial magnetic stimulation has been widely used at the cortex level to investigate its effects on motor excitability. However, it has been reported that transcranial magnetic stimulation generally induces a differential response between left‐ and right‐side muscles. For example, Khedr and Trakhan (2001) demonstrated that transcranial magnetic stimulation above the left hemisphere induced a greater motor‐evoked potential in the right diaphragm than in the left diaphragm. Similarly, a higher amplitude signal is usually observed in the contralateral biceps than in the ipsilateral biceps in response to transcranial magnetic stimulation (Bawa et al., 2004). The differential response between the contralateral and ipsilateral muscles is primarily due to a difference in the proportions of crossed and uncrossed corticospinal tracts. When the coil is moved down to the spinal cord, stimulation at this position has been shown to induce activation of the bilateral muscles in both human and animal studies (Lee et al., 2021, 2022; Ren et al., 2022; Urban et al., 2002). The ability of trans‐spinal magnetic stimulation to co‐activate bilateral muscles is mainly due to the fact that the magnetic field can cover the whole spinal cord and/or bilateral spinal roots/nerves. This characteristic of trans‐spinal magnetic stimulation can be used to examine the differential response of bilateral muscles under physiological or pathological conditions.

Additionally, we found that the excitability in the right‐side muscle is greater than that in the left‐side muscle. The difference may be contributed to handedness, as almost all participants (18 participants) are right‐handed. Several factors, such as lower recruitment threshold of motor units, higher firing rate of motor units (Adam et al., 1998), greater motoneuron excitability (Tan, 1989), and higher composition of type I muscle fiber (Fugl‐Meyer et al., 1982), are typically found in the dominant hand compared to the nondominant hand. Accordingly, trans‐spinal magnetic stimulation may likely induce a greater response in the dominant side muscles (i.e., right side). A similar result was also observed in previous studies (Spiesshoefer et al., 2019; Zifko et al., 1996).

### 4.3 Co‐activation of the diaphragm and biceps during trans‐spinal magnetic stimulation

In addition to the co‐activation of bilateral muscles, the present study demonstrated that a single pulse of trans‐spinal magnetic stimulation using a figure‐of‐eight coil with two wings covering the mid‐cervical to upper thoracic portion of the spinal cord can comprehensively activate the diaphragm and biceps. The magnetic field generated by this stimulatory configuration can cover a longitudinal region of the spinal cord and then stimulate the motoneurons and spinal roots/nerves from the mid‐cervical to high‐thoracic level. Although there would be three hot spots (i.e., the center of the coil and the centers of the two wings) inducing greater eddy currents within the spinal cord, this approach can effectively activate both respiratory and forelimb muscles simultaneously. The co‐activation of multiple muscles using a single trans‐spinal magnetic stimulatory approach may be more efficient and convenient than magnetic stimulation of the cortex or peripheral nerves.

The results of the present study demonstrate that the responsiveness of the biceps is greater than that of the diaphragm during trans‐spinal magnetic stimulation in males. The mechanism underlying this difference requires further investigation. We have proposed several factors that may contribute to the differential response of the biceps and the diaphragm. First, the phrenic nucleus innervating the diaphragm in humans are primarily located in the third to fifth cervical spinal cord (Routal & Pal, 1999), while the biceps is innervated by motoneurons within third or fifth to seventh spinal cord (Schirmer et al., 2011; Waxenbaum et al., 2022). The larger area of the biceps motoneuron pool and/or the larger number of nerve roots may be responsible for the greater susceptibility to trans‐spinal magnetic stimulation observed in the biceps relative to the diaphragm. In addition, the center of the stimulus coil is closer to biceps motoneurons, roots, and nerves. It may also be a reason for the greater response in biceps than in the diaphragm. Second, the cross‐sectional area of the diaphragm fiber (2225.5 ± 130.6 to 2502.6 ± 225.8 μm^2^, mean ± SE) is smaller than that of the biceps (5261 ± 1211 μm^2^, mean ± SD) (Duchateau & Enoka, 2022; Meznaric & Cvetko, 2016). The smaller diaphragm fiber size results in a lower amplitude of motor unit potential compared to the biceps (Podnar & Resman‐Gaspersic, 2008), and this may lead to a weaker motor‐evoked potential during trans‐spinal magnetic stimulation. Third, the composition of muscle fiber is also different between the diaphragm and the biceps. For example, a previous study indicated that the adult human diaphragm consists of approximately 55% slow‐twitch and 45% fast‐twitch fibers (Polla et al., 2004), while the biceps are composed of approximately 39% slow‐twitch and 61% fast‐twitch fibers (Dahmane et al., 2005). As slow‐twitch fibers are usually smaller than fast‐twitch fibers and have a weaker motor unit potential, diaphragmatic motor‐evoked potentials are expected to be smaller than biceps motor‐evoked potentials. Fourth, the surface electrodes used for the diaphragm EMGs were placed on the low intercostal space and were not directly attached to the costal diaphragm. This configuration may attenuate volume conduction and dampen the intensity of the signal generated by the diaphragm. However, the surface electrodes were in direct contact with the biceps, which may have enabled the electrodes to receive robust signals from the biceps.

### 4.4 Effectiveness of trans‐spinal magnetic stimulation using a figure‐of‐eight coil with a different orientation

Our previous report demonstrated that trans‐spinal magnetic stimulation using a figure‐of‐eight coil with two wings covering the bilateral spinal roots/nerves can induce motor‐evoked potentials bilaterally in the diaphragm (Ren et al., 2022). However, diaphragmatic motor‐evoked potential amplitude induced by this stimulatory configuration is weaker than the response induced by a figure‐of‐eight coil with two wings covering the mid‐cervical to high‐thoracic portion of the spinal cord in this study. This differential response is mainly caused by differences in the coverage of the electromagnetic field and suggests that the current study established a more effective trans‐spinal magnetic stimulation to induce co‐activation of bilateral diaphragm and biceps.

### 4.5 Sex effect

Two differences in the responses of male and female subjects were observed in this study. First, a stronger response was observed in the biceps than in the diaphragm during trans‐spinal magnetic stimulation in the right side of males. Second, a significantly stronger response was observed in the right biceps than the left biceps in males but not females. Our results demonstrated that the biceps response is generally weaker in females, and female subjects exhibited a similar response between the left and right biceps, and between the diaphragm and biceps. A previous study showed that men were found to have significantly larger type I fiber area (4597 vs. 3483 μm^2^) and mean fiber area (6632 vs. 3963 μm^2^) than women in the biceps brachii (Miller et al., 1993). This anatomic difference may be the cause of the lower biceps motor‐evoked potentials observed in female subjects, as well as the fact that we were unable to reveal any difference between the responses in the biceps and the diaphragm during trans‐spinal magnetic stimulation.

## 5 CONCLUSION

This study demonstrated that trans‐spinal magnetic stimulation using a figure‐of‐eight coil is a feasible and efficient approach to co‐activate the bilateral diaphragm and biceps. Although the responses to trans‐spinal magnetic stimulation may exhibit a mild asymmetry between muscles on the left and right sides due to anatomical factors, the co‐activation of respiratory and forelimb muscles in response to trans‐spinal magnetic stimulation provides a proof of concept for developing a strategy to activate respiratory and forelimb muscles in the future.

## CONFLICT OF INTEREST STATEMENT

There are no conflicts of interests for all authors.

## ETHICS STATEMENT

All experimental procedures were approved by the Institutional Review Board at Kaohsiung Medical University Hospital (KMUHIRB‐F(I)‐20210039). This study was conducted in accordance with the Declaration of Helsinki.
